# Chlortetracycline and florfenicol induce expression of genes associated with pathogenicity in multidrug-resistant *Salmonella enterica* serovar Typhimurium

**DOI:** 10.1186/s13099-018-0236-y

**Published:** 2018-03-05

**Authors:** Devin B. Holman, Shawn M. D. Bearson, Bradley L. Bearson, Brian W. Brunelle

**Affiliations:** 1 Food Safety and Enteric Pathogens Research Unit, National Animal Disease Center, ARS, USDA, Ames, IA USA; 2 Agroecosystems Management Research Unit, National Laboratory for Agriculture and the Environment, ARS, USDA, Ames, IA USA; 3 Present Address: Lacombe Research and Development Centre, Agriculture and Agri-Food Canada, Lacombe, AB Canada; 4 Present Address: Arbor Biosciences, Ann Arbor, MI USA

**Keywords:** Multidrug-resistant, *Salmonella*, Florfenicol, Chlortetracycline, RNA-seq, Invasion, Motility

## Abstract

**Background:**

Multidrug-resistant (MDR) *Salmonella enterica* serovar Typhimurium (*S*. Typhimurium) is a serious public health threat as infections caused by these strains are more difficult and expensive to treat. Livestock serve as a reservoir for MDR *Salmonella*, and the antibiotics chlortetracycline and florfenicol are frequently administrated to food-producing animals to treat and prevent various diseases. Therefore, we evaluated the response of MDR *S*. Typhimurium after exposure to these two antibiotics.

**Results:**

We exposed four MDR *S*. Typhimurium isolates to sub-inhibitory concentrations of chlortetracycline (16 and 32 µg/ml) or florfenicol (16 µg/ml) for 30 min during early-log phase. Differentially expressed genes following antibiotic treatment were identified using RNA-seq, and genes associated with attachment and those located within the *Salmonella* pathogenicity islands were significantly up-regulated following exposure to either antibiotic. The effect of antibiotic exposure on cellular invasion and motility was also assessed. Swimming and swarming motility were decreased due to antibiotic exposure. However, we observed chlortetracycline enhanced cellular invasion in two strains and florfenicol enhanced invasion in a third isolate.

**Conclusions:**

Chlortetracycline and florfenicol exposure during early-log growth altered the expression of nearly half of the genes in the *S*. Typhimurium genome, including a large number of genes associated with virulence and pathogenesis; this transcriptional alteration was not due to the SOS response. The results suggest that exposure to either of these two antibiotics may lead to the expression of virulence genes that are typically only transcribed in vivo, as well as only during late-log or stationary phase in vitro.

**Electronic supplementary material:**

The online version of this article (10.1186/s13099-018-0236-y) contains supplementary material, which is available to authorized users.

## Background

*Salmonella* is a leading cause of bacterial foodborne disease in humans, resulting in an estimated 94 million cases worldwide and 1 million cases in the United States every year. Livestock are often asymptomatically colonized by *Salmonella*, with 40–50% of US beef cattle and swine operations testing positive [1–3]. *Salmonella* isolated from food-producing animals are frequently multidrug-resistant (MDR; resistant to ≥ 3 or more antimicrobial classes), with *Salmonella enterica* serovar Typhimurium (*S*. Typhimurium) being the most common MDR serovar identified; in fact, nearly half of all MDR *Salmonella* isolates over a 12-year span in the United States were *S*. Typhimurium. Of note is the high level of resistance to ampicillin (69.9%), chloramphenicol (56.3%), streptomycin (72.1%), and tetracycline (87.6%) found in *S*. Typhimurium isolated from swine over this 12-year period [4].

We previously established that invasion gene expression and bacterial invasion was induced in several MDR *S*. Typhimurium strains following exposure to sub-inhibitory levels of tetracycline and chloramphenicol for 30 min during the non-invasive early-log growth phase [5]. Resistance to tetracycline and chloramphenicol is mediated by the specific efflux pumps *tetA/G* and *floR* in these MDR *S*. Typhimurium strains, respectively. Interestingly, one particular MDR isolate that encoded *tetBCD* and the chloramphenicol-inactivation enzyme, *cat*, did not have significantly different gene expression profiles or invasion phenotypes due to either antibiotic. In addition, exposure to ampicillin and streptomycin with enzyme-mediated resistance mechanisms did not affect invasion phenotypes nor did the antibiotics change the expression of the invasion pathway regulating the *hilA* gene (as observed with tetracycline and chloramphenicol). Thus, it is possible that efflux-encoded resistance mechanisms are required for the considerable transcriptomic and phenotypic differences that were observed.

Chlortetracycline and florfenicol are analogues of tetracycline and chloramphenicol, respectively, and are commonly used in veterinary medicine at concentrations that can be considered sub-inhibitory to MDR strains but lethal to sensitive isolates. Given the high incidence of *Salmonella* in livestock, especially those that are MDR, we characterized the effect that chlortetracycline and florfenicol had on several MDR *S*. Typhimurium isolates. We found that exposure to sub-inhibitory concentrations of either antibiotic led to over 50% of the genes in the genome being differentially regulated, resulting in changes in invasion and motility phenotypes.

## Methods

### *Salmonella* isolates and growth conditions

Four isolates of *Salmonella enterica* serovar Typhimurium phage types DT104 (530) and DT193 (1306, 1434, and 5317) originally collected from cattle were selected for this study based on previous research [5, 6]. These isolates are resistant to ampicillin, chloramphenicol, chlortetracycline (CTC), florfenicol, streptomycin, and tetracycline, but are sensitive to gentamicin (Additional file 1: Table S1).

To prepare overnight cultures used in the various assays, each of the four *Salmonella* isolates were streaked onto separate solid LB agar plates (Invitrogen, Carlsbad, CA, USA). Individual colonies were picked and grown at 37 °C in LB broth with agitation for 6 h. A 1:1000 dilution in LB broth was grown at 37 °C overnight with agitation.

### *Salmonella* growth curves

To determine the concentrations of CTC and florfenicol that inhibited growth of the four *Salmonella* isolates, growth curves were performed using the same conditions utilized for gene expression analysis. Overnight cultures were diluted 1:200 in LB broth and grown to early-log phase (OD_600_ = 0.15) at 37 °C with agitation. The samples were aliquoted into 100-well plates, and serial twofold dilutions of CTC or florfenicol (0, 2–512 µg/ml) were added to the appropriate wells. Using the Bioscreen C Growth Curve machine (Growth Curves LTD, Raisio, Finland), the cultures were shaken continuously at 37 °C and growth measurements (OD_600_) were taken every hour for 24 h. All conditions were performed in triplicate.

### RNA extraction and RNA-sequencing

Overnight cultures were diluted 1:200 in LB and grown to an OD_600_ of 0.15 at 37 °C with agitation. CTC and florfenicol were added at the indicated concentrations, and the cultures were incubated at 37 °C with agitation for 30 min. One aliquot of each culture was placed in RNA Protect (Qiagen, Germantown, MD, USA) according to manufacturer’s directions. A second aliquot from each sample was used immediately in an invasion assay (see below). RNA was extracted from cell pellets preserved in RNA Protect (Qiagen) using the RNeasy Mini Kit (Qiagen) followed by treatment with Turbo DNase (Ambion, Austin, TX, USA) to remove genomic DNA. Total RNA was evaluated using a 2100 Bioanalyzer (Agilent Technologies, Santa Clara, CA, USA). The Ribo-Zero rRNA removal kit (Illumina, Inc., San Diego, CA, USA) was used to deplete the bacterial ribosomal RNA sequences per the manufacturer’s instructions, and the 2100 Bioanalyzer was used to assess the quality of the rRNA removal step. Libraries were constructed using TruSeq Stranded Total RNA Library prep kits (Illumina, Inc.) and were sequenced at the Iowa State University DNA facility on an Illumina HiSeq 2500 (100 cycles).

### Analysis of RNA-sequencing data

Reads were aligned to the *S*. Typhimurium SL1344 genome (GenBank Accession Number FQ312003.1), its three plasmids (GenBank Accession Numbers HE654724-26), and the MDR *S*. Heidelberg plasmid psSH163_35 (GenBank Accession Number JN983045) using Bowtie 2 v. 2.2.9 [7] with the default parameters for the very-sensitive-local setting. The program HTSeq v. 0.6.1 [8] was used to quantify the number of reads that aligned to a specific gene within the *S*. Typhimurium SL1344 genome and the plasmids for each *S*. Typhimurium strain and antibiotic concentration used. Differentially expressed genes (false discovery rate [FDR] of 0.05) among the different antibiotic concentrations were identified using the DESeq 2 package v. 1.14.1 [9] in R v. 3.4.0. Principal component analysis (PCA) plots were generated based on the regularized log-transformed counts for each *S*. Typhimurium strain, antibiotic, and antibiotic concentration. Genes that were either significantly differentially over- or under-expressed were submitted to the PANTHER classification system v. 11.1 (http://pantherdb.org/) [10] for classification into gene ontology (GO) terms. The significant enrichment of specific GO terms was determined for each strain and antibiotic treatment using Bonferroni corrected P-values. All RNA-seq files were deposited in the Sequence Read Archive (https://www.ncbi.nlm.nih.gov/sra) under BioProject accession PRJNA344670.

### Motility assays

A 1:200 dilution of an overnight culture was grown to an OD_600_ of 0.3 at 37 °C with agitation. The media for the swimming (0.3% agar LB) and swarming (0.5% agar LB with 0.5% glucose) motility assays were made as previously described [11, 12]. Briefly, the motility media was autoclaved and then allowed to reach equilibrium in a 56 °C water bath, after which antibiotics and glucose (swarm only) were added and poured into 100 × 15 mm culture plates and allowed to solidify. A 5 µl aliquot of each *Salmonella* isolate (OD_600_ = 0.3) was pipetted onto the center of each plate, left on the bench for 10 min (covered), and then incubated at 37 °C for ~ 5 h (swim) or ~ 10 h (swarm). Two technical and three biological replicates were performed for each isolate and antibiotic concentration. The diameters of the bacterial swimming and swarming motility were measured and normalized to the no-antibiotic control.

### Invasion assays

After the 30 min antibiotic incubation performed above (see “RNA extraction and RNA-sequencing” section), the aliquot taken from each sample was immediately centrifuged at 16,000×*g* for 2 min, decanted, and resuspended in fresh LB in order to remove the antibiotic. Invasion assays (i.e. gentamicin protection assays) were performed as previously described [13] in HEp-2 cells using a multiplicity of infection of ~ 40. Invasion was calculated as the CFU/ml recovered from the cells divided by the CFU/ml added to the cell culture. All invasion assays included technical replicates and were repeated three times.

### Statistical analysis

The R package lme4 v 1.1.12 [14] was used to assess the effect of CTC and florfenicol on *S*. Typhimurium invasion and motility for each strain. A linear mixed model was used with replication as the random effect and antibiotic concentration as the fixed effect. Post-hoc comparisons against the untreated control were carried out using the R package lsmeans 2.25.5 [15] and the Dunnett adjustment.

## Results and discussion

Many of the genes associated with virulence in *S*. Typhimurium are encoded within at least five *Salmonella* pathogenicity islands (SPI), which are clusters of virulence genes that are required to facilitate host cell entry and intracellular survival [16, 17]. These virulence genes are temporally regulated along with motility and attachment genes [17–19]. In liquid culture during early-log growth, *Salmonella* display little-to-no invasiveness as motility expression is up-regulated while SPI and attachment gene expression are down-regulated. We previously demonstrated that the virulence regulation pathway that can take 8–12 h in culture can be induced in 30 min in select MDR *S*. Typhimurium isolates by exposure to either chloramphenicol or tetracycline [5]. In the present study, we used transcriptional and phenotypic assays to determine the similarities and differences in the early-log growth regulation in response to the veterinary antibiotics chlortetracycline and florfenicol.

### Growth characteristics of MDR *S*. Typhimurium strains exposed to chlortetracycline or florfenicol

Growth curves were generated for all four MDR *S*. Typhimurium strains following exposure to serial two-fold dilutions of CTC or florfenicol to determine the minimum inhibitory concentrations for each isolate and antibiotic (Additional file 1: Table S1). At a concentration of 128 µg/ml, CTC inhibited the growth of strain 530, while all strains were inhibited at 256 µg/ml. All strains were completely inhibited by 64 µg/ml of florfenicol with strain 530 also being inhibited by 32 µg/ml.

### Effect of chlortetracycline and florfenicol exposure on the transcriptome of MDR *S*. Typhimurium

**Fig. 1:**
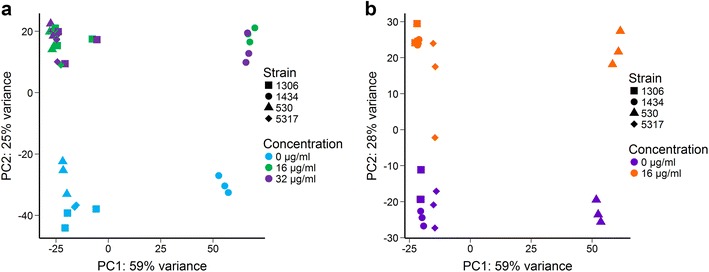
Principal component analysis (PCA) plot of the regularized log-transformed counts for each MDR *S*. Typhimurium strain following exposure to **a** chlortetracycline or **b** florfenicol. The percentages of variation explained by each component is displayed on the axes

Based on the growth curve analysis, 16 and 32 µg/ml of CTC and 16 µg/ml of florfenicol were used to assess the impact these antibiotics have on influencing the transcriptome and the invasive phenotype of the four MDR *S*. Typhimurium isolates. There were 14,539,717 ± 338,608 SEM (standard error of the mean) reads per sample for the CTC experiment and 16,917,718 ± 376,379 SEM for florfenicol. On average, the exposure of the four *S*. Typhimurium isolates to either CTC or florfenicol resulted in the significant differential expression of 3334 ± 109 and 3126 ± 377 genes (SEM; FDR < 0.05), respectively. These values represent 62 and 58% of the total genes in the *S*. Typhimurium SL1344 genome and plasmids analyzed, respectively. Among these genes, 1487 were differentially expressed in all strains at the highest concentration of both antibiotics (FDR < 0.05). Principal component analysis (PCA) plots of the normalized gene counts demonstrated a relatively large shift in the transcriptome of all *S*. Typhimurium strains exposed to either CTC (Fig. 1a) or florfenicol (Fig. 1b). For the CTC exposed strains, both 16 and 32 µg/ml of CTC altered the transcriptome to a similar degree. Interestingly, strain 1434 clustered separately from the other three strains at all CTC concentrations (i.e. 0, 16, and 32 µg/ml) while strain 530 clustered separately when exposed to florfenicol at 0 or 16 µg/ml. A summary of the fold changes with gene groups of interest for each isolate and antibiotic is displayed in Table 1.

**Table 1:** Mean fold changes (± standard error of the mean) for genes within each functional gene group following exposure to either chlortetracycline at 32 µg/ml or florfenicol at 16 µg/ml for 30 min

Gene group	Chlortetracycline				Florfenicol			
	530	1306	1434	5317	530	1306	1434	5317
Attachment	4.8 ± 0.7	5.6 ± 0.9	13.2 ± 2.4	10.6 ± 1.9	4.3 ± 0.5	3.9 ± 0.5	7.1 ± 0.8	3.8 ± 0.3
Efflux	1.9 ± 0.9	1.1 ± 0.9	3.2 ± 1.5	2.8 ± 1.7	1.6 ± 0.7	0.7 ± 0.6	1.5 ± 0.8	0.9 ± 0.7
Motility	− 10.3 ± 1.4	− 7.5 ± 1.0	− 10.6 ± 1.4	− 9.2 ± 1.0	− 10.9 ± 1.4	− 7.9 ± 1.1	− 9.3 ± 1.3	− 4.2 ± 0.4
SOS response	0.6 ± 0.3	0.8 ± 0.4	1.0 ± 0.4	1.2 ± 0.5	0.3 ± 0.4	0.6 ± 0.4	0.5 ± 0.4	0.6 ± 0.4
SPI-1	1.4 ± 0.4	2.3 ± 0.4	13.7 ± 1.9	10.9 ± 1.1	0.1 ± 0.5	0.5 ± 0.4	6.5 ± 0.9	4.5 ± 0.5
SPI-2	5.4 ± 0.8	5.6 ± 1.0	16.7 ± 3.3	12.3 ± 2.3	3.6 ± 0.7	4.3 ± 0.7	9.6 ± 2.0	4.5 ± 0.7
SPI-3	15.8 ± 11.9	7.8 ± 4.0	23.6 ± 17.8	23.1 ± 15.9	9.9 ± 7.4	3.4 ± 1.8	11.9 ± 8.1	11.5 ± 8.5
SPI-4	− 0.2 ± 0.7	0.7 ± 0.7	14.6 ± 5.9	14.3 ± 4.0	− 1.3 ± 1.1	− 3.0 ± 1.5	2.0 ± 0.3	1.5 ± 0.1
SPI-5	3.6 ± 2.9	2.2 ± 3.6	20.2 ± 12.9	9.8 ± 8.2	2.9 ± 3.0	1.2 ± 3.2	6.9 ± 5.5	2.6 ± 1.9

### Attachment genes

**Fig. 2:**
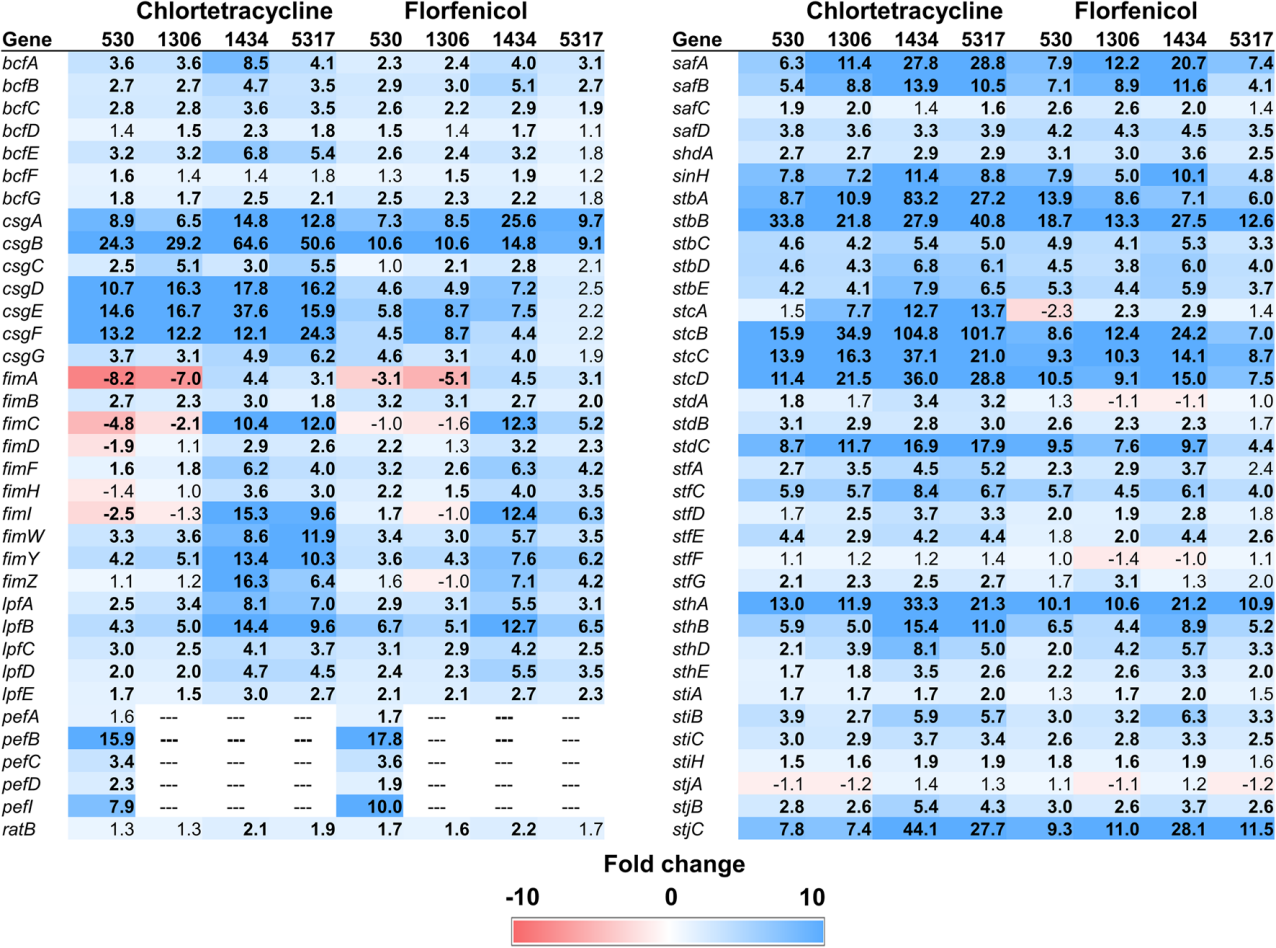
Fold changes for attachment-associated genes following exposure to either chlortetracycline at 32 µg/ml or florfenicol at 16 µg/ml for 30 min. Numbers in bold indicate significantly differentially expressed genes (FDR < 0.05). Genes that are up-regulated are colored blue while down-regulated genes are in red; the intensity of the color indicates greater fold change

Seventy genes related to *S*. Typhimurium attachment were evaluated for differential gene expression (Fig. 2). Of these genes, 37 were significantly over-expressed among all strains exposed to either antibiotic at the highest concentration used. Exposure to 16 µg/ml of florfenicol resulted in a consistent up-regulation (up-regulated in at least three strains) of 53 attachment-related genes in the MDR *S*. Typhimurium isolates. In addition, 55 attachment-related genes were consistently up-regulated following exposure to 32 µg/ml of CTC. Among these were the curli-associated genes, *csgABDEF*, and fimbrial genes, namely *stbABCDE* and *stcABCD*. Another fimbrial gene, *safA* (*Salmonella* atypical fimbriae lipoprotein), was also highly up-regulated among all strains. Notably, *safA* has been shown to be important in the colonization of the ileum in pigs [20]. However, an in vitro study by Kroger et al. [21] reported low expression levels for *safA* in *S*. Typhimurium at all growth phases tested, even when exposed to a variety of different environmental stress conditions. Similarly, these authors observed little to no expression of the *csgABDEF*, *stcABCD* and *stbABCDE* genes under any of the conditions or growth phases investigated [21]. Therefore, it appears that exposure to either CTC or florfenicol results in gene expression for MDR *S*. Typhimurium that is typically only found in sensitive strains in vivo [22]. With the exception of the *fim* genes, some of which were under-expressed in strains 530 and 1306, the same attachment-associated genes tended to be up-regulated in both CTC and florfenicol exposed cells. Only *S*. Typhimurium strain 530 had significantly higher expression of the *pefBCDI* (plasmid-encoded fimbriae) genes when exposed to CTC and florfenicol as the *pefABCDI* genes are absent in strains 1306, 1434, and 5317.

Typically, the *fim* genes are expressed in liquid culture during the stationary growth phase in *Salmonella*, while the *csg* and *pef* genes require solid or low pH growth media, respectively [21]. Similar to our previous experiment [5], the *csg* and *pef* genes (when present), as well as other attachment genes that are usually only up-regulated in a host, were induced by CTC and florfenicol. Of note, *stb*, *stc*, *std*, and *sth* genes, which are necessary for persistent *Salmonella* infection in mice [22], were induced by antibiotic exposure.

### SPI-1

**Fig. 3:**
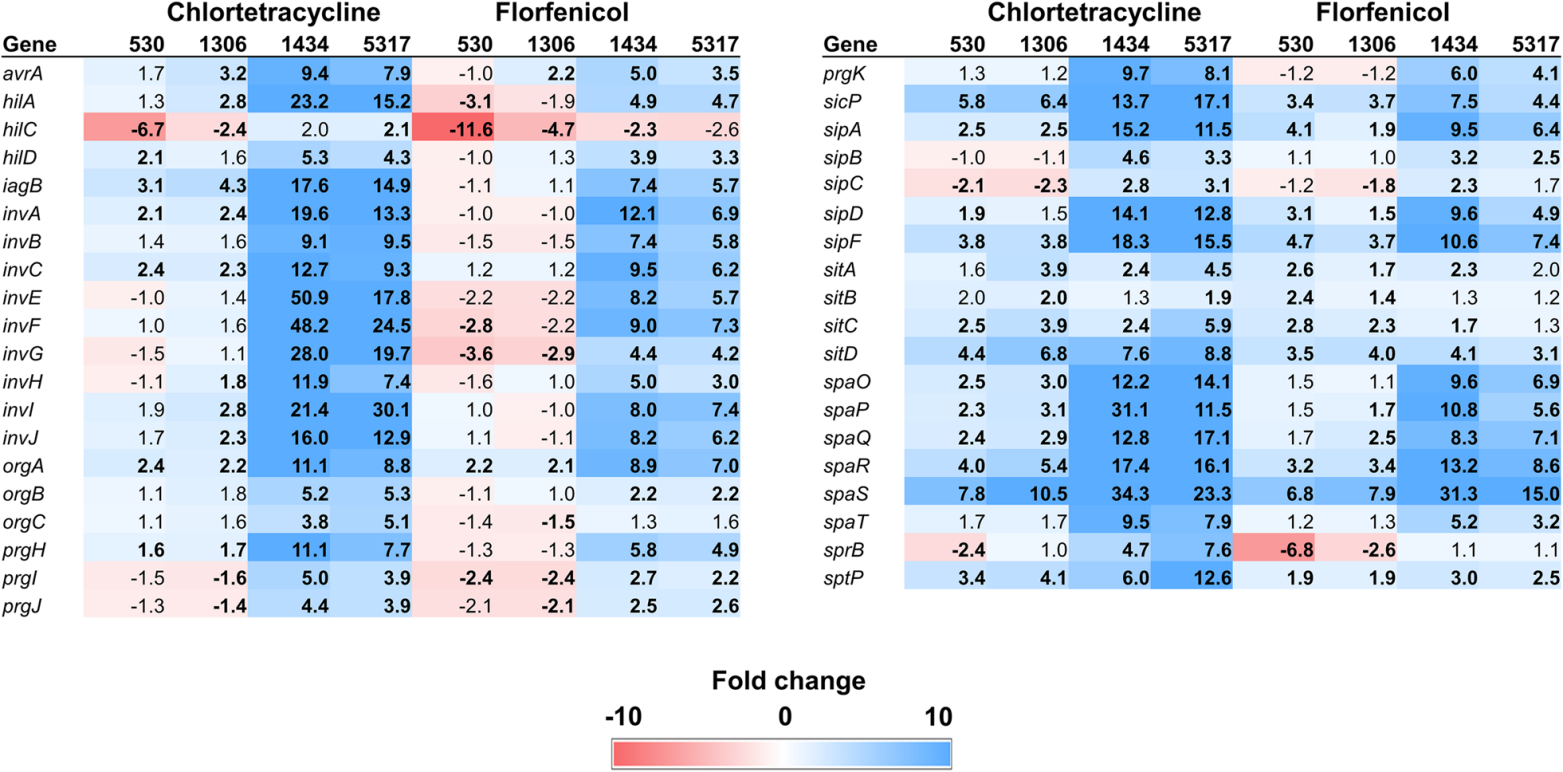
Fold changes for SPI-1 genes following exposure to either chlortetracycline at 32 µg/ml or florfenicol at 16 µg/ml for 30 min. Numbers in bold indicate significantly differentially expressed genes (FDR < 0.05). Genes that are up-regulated are colored blue while down-regulated genes are in red; the intensity of the color indicates greater fold change

*Salmonella* pathogenicity island-1 (SPI-1) contains 39 genes, many of which are involved in the invasion of host epithelial cells [16]. Interestingly, nearly all the SPI-1 genes in isolates 1434 and 5317 were up-regulated; however, only *spaS* in isolates 530 and 1306 had an increase in expression of greater than sixfold (Fig. 3). This observation is very similar to those of a previous study where three of these isolates were exposed to tetracycline or chloramphenicol at 16 and 64 µg/ml, respectively [5]. In isolates 1434 and 5317, the *invABCEFGHIJ*, *prgHIJK*, and *spaOPQRST* genes were up-regulated with both CTC and florfenicol. Genes within these three operons, along with the *org* operon, encode for proteins that form the type III secretion apparatus (T3SS-1) [17], and it is this T3SS-1 apparatus that is involved in the invasion of host cells [23].

### SPI-2

**Fig. 4:**
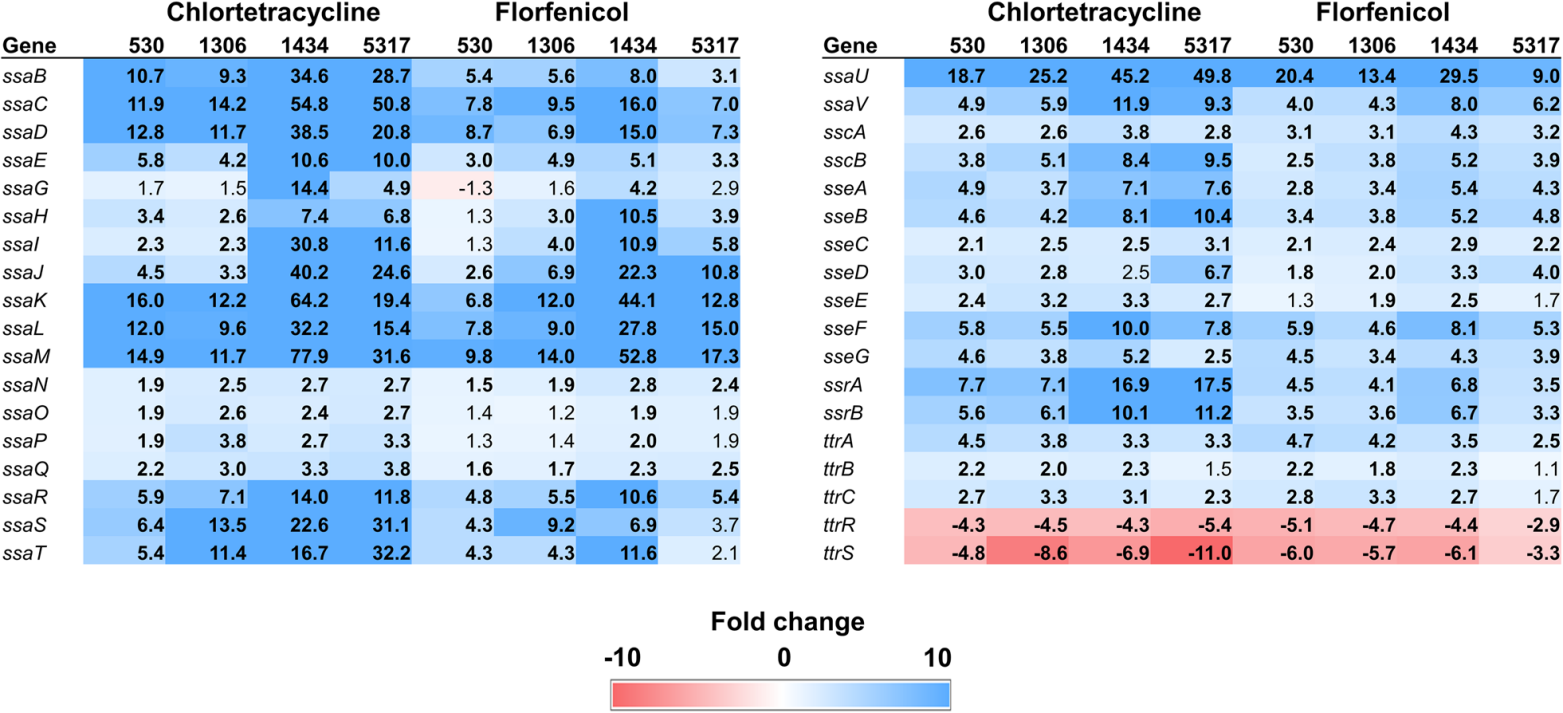
Fold changes for SPI-2 genes following exposure to either chlortetracycline at 32 µg/ml or florfenicol at 16 µg/ml for 30 min. Numbers in bold indicate significantly differentially expressed genes (FDR < 0.05). Genes that are up-regulated are colored blue while down-regulated genes are in red; the intensity of the color indicates greater fold change

At least 36 genes are found within SPI-2, including the *ssa* (secretion system apparatus) genes that encode for the structural proteins of the T3SS-2 apparatus [24]. The T3SS-2 and its effector proteins (encoded by the *sse* genes) contribute to the intracellular survival of *S*. Typhimurium in the host [23]. In the present study, a majority of the *ssa* and *sse* genes were significantly up-regulated in all isolates exposed to either CTC or florfenicol (Fig. 4). Strains 1434 and 5317 were again most strongly affected by CTC exposure in terms of SPI-2 gene expression. Only *ttrR* and *ttrS*, which are part of a two-component regulatory system for tetrathionate reduction [25], had significantly lower expression levels among all strains following antibiotic exposure. Antibiotic sensitive *S*. Typhimurium cells grown in LB broth under normal conditions to early-, mid-, or late-log phase typically express very few of the SPI-2 genes [21]. This once again demonstrates that in MDR *S*. Typhimurium isolates, exposure to chlortetracycline or florfenicol results in the expression of genes that are not normally transcribed in vitro in the absence of environmental stressors.

### SPI-3

**Fig. 5:**
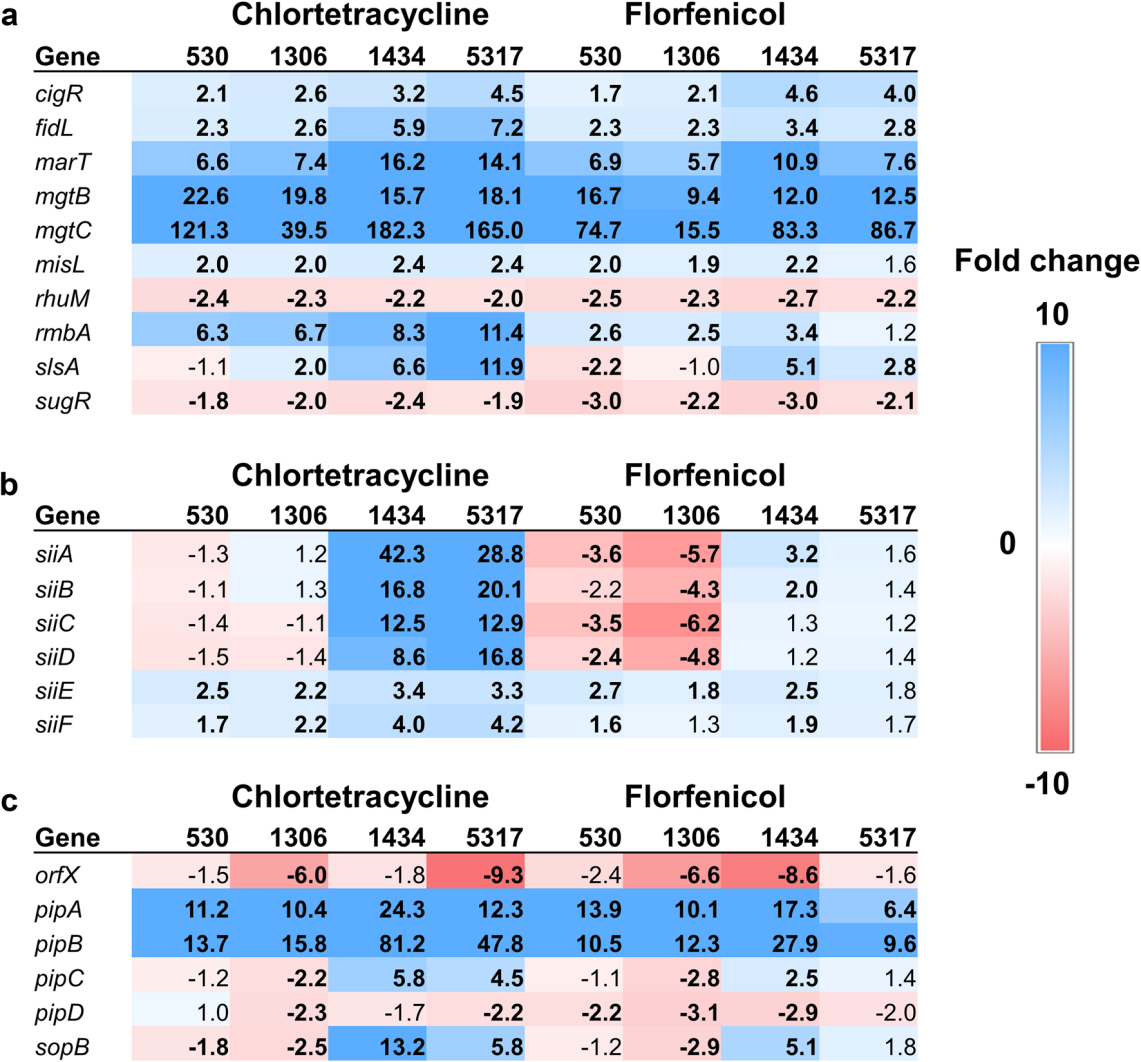
Fold changes for **a** SPI-3, **b** SPI-4, and **c** SPI-5 genes following exposure to either chlortetracycline at 32 µg/ml or florfenicol at 16 µg/ml for 30 min. Numbers in bold indicate significantly differentially expressed genes (FDR < 0.05). Genes that are up-regulated are colored blue while down-regulated genes are in red; the intensity of the color indicates greater fold change

The number of genes encoded within SPI-3, SPI-4, and SPI-5 is considerably fewer than either SPI-1 or SPI-2 [17]. Among the SPI-3 genes, *cigR*, *fidL*, *marT, mgtB*, and *mgtC* were significantly over-expressed among all isolates and with both antibiotics (Fig. 5a). Specifically, *mgtC* was one of the most highly up-regulated genes in the entire transcriptome following antibiotic exposure. This is in strong agreement with our earlier study when MDR *S*. Typhimurium strains exposed to either tetracycline or chloramphenicol had significantly increased expression of both *mgtB* and *mgtC* [1]. The *mgtB* gene encodes for a magnesium transporter [26] and *mgtC* encodes for a membrane protein of unknown function but is required for survival in macrophages and in low Mg^2+^ concentrations in liquid media [27, 28]. Carnell et al. [20] reported that a *S*. Typhimurium strain with a mutation in *mgtC* exhibited attenuated colonization in the ileum of pigs.

### SPI-4

The SPI-4 region contains only six genes, the *siiABCDEF* operon [17]. The *siiCDF* genes encode a type I secretion system (T1SS) apparatus, and SiiE is a giant non-fimbrial adhesion protein that is secreted through the T1SS apparatus [29, 30] and is associated with intestinal colonization in cattle [31]. The *siiA* and *siiB* genes encode membrane-associated proteins [30]. All of the genes within SPI-4 were significantly up-regulated in isolates 1434 and 5317 but only following CTC treatment (Fig. 5b). However, there was little-to no expression changes for the *sii* genes in these strains after florfenicol exposure, and in strains 530 and 1306, genes *siiABCD* were significantly down-regulated by florfenicol. Typically, these genes are not expressed until late-log phase under normal growth conditions [21].

### SPI-5

At least six genes are encoded within the SPI-5 region (*pipABCD*, *sopB*, and *orfX*), and these genes are associated with enteropathogenesis [32]. Exposure to either CTC or florfenicol significantly increased expression of *pipA* and *pipB* in all four of the strains evaluated. Notably, the *pipB* gene was among the most highly up-regulated genes in isolates 1434 and 5317 following CTC treatment (Fig. 5c). PipB is an SPI-2 effector protein that is translocated through the T3SS-2 apparatus [33]. Previously, *S*. Typhimurium cells treated with either tetracycline or chloramphenicol also displayed significantly higher expression of *pipB* [5]. The *pipC* (*sigE*) gene encodes a chaperone protein for SopB (SigD) [34], which is an inositol phosphatase, and expression of both these genes followed a similar pattern; however, these genes were only significantly up-regulated in strains 1434 and 5317 following CTC exposure and in 1434 when exposed to florfenicol. A mutation in the *pipC* gene has been shown to reduce colonization in the ileum of *S*. Typhimurium in pigs [20].

### Motility and SOS response genes

In our previous study, a large number of genes associated with motility were significantly down-regulated when MDR *S*. Typhimurium strains were exposed to either chloramphenicol or tetracycline [5]. In the current study, we replicated this finding with chlortetracycline and florfenicol as the majority of the *flg* and *fli* genes had a significant reduction in expression following antibiotic exposure (Additional file 2: Figure S1). The transcriptomic analysis also reflected the phenotypic motility as both swarming and swimming were significantly diminished following antibiotic treatment (P < 0.05; Additional file 3: Figure S2). In LB broth, the expression of *flg* and *fli* genes in antibiotic sensitive *S*. Typhimurium strains is lowest during the stationary phase [21].

Survival and persistence of bacteria following exposure to subinhibitory antibiotic concentrations is often attributed to the SOS response [35–38]. However, differential gene expression for genes associated with the SOS response did not indicate a coordinated response following exposure to CTC or florfenicol (Additional file 4: Figure S3). For example, *recA* and *lexA* were either down-regulated or the change was not significant, respectively. However, gene expression for *dbh*, *symE*, *umuC*, and *uvrB* was significantly increased, and *dinF* was significantly decreased in all isolates by both antibiotics. This suggests that the significant up-regulation of invasion and attachment genes was not related to the SOS response. It has been reported that in *Salmonella,* the SOS response, and in particular the expression of *recA*, can lead to decreased swarming in antibiotic-sensitive isolates [39]. However, the lack of a coordinated SOS response that is coincident with the observed decrease in the motility phenotype due to antibiotic exposure suggests an alternative mechanism in the isolates for the current study.

### Gene ontology analysis of differentially expressed genes

All of the genes that were either over- or under-expressed following antibiotic exposure were classified into functional groups based on gene ontology (GO) terms. Among the genes that were up-regulated, genes in the transmembrane transport category (as well as in the related transport, establishment of localization, and localization GO terms) were significantly enriched in all strains following exposure to either antibiotic (Table 2). There are 420 genes associated with this GO term in *S*. Typhimurium including the *mgt* and *sse* genes. Genes within the pathogenesis and cell adhesion categories were also enriched in strains 1434 and 5317 and with both antibiotic treatments. This is expected given that the pathogenesis GO term encompasses several highly up-regulated SPI genes such as *invABCEFGHIJ*, *spaOPQRST*, *ssaBELR*, *sseABCIJL*, and *pipB*. Genes that were down-regulated were enriched for several metabolic and motility GO terms (Additional file 5: Table S2).

**Table 2:** Gene ontology (GO) terms describing biological processes that were enriched among the significantly differentially over-expressed genes in each *S*. Typhimurium strain and antibiotic treatment

Gene ontology term	Chlortetracycline				Florfenicol			
	530	1306	1434	5317	530	1306	1434	5317
Cell adhesion (GO:0007155)	NS	NS	2.6	2.4	NS	NS	2.3	2.8
Biological adhesion (GO:0022610)	NS	NS	2.6	2.4	NS	NS	2.3	2.8
Multi-organism process (GO:0051704)	1.7	NS	NS	NS	NS	NS	NS	NS
Pathogenesis (GO:0009405)	NS	1.8	2.3	2.2	NS	NS	2.1	3.0
Interspecies interaction between organisms (GO:0044419)	NS	1.8	2.1	2.0	NS	NS	1.9	2.6
Multi-organism process (GO:0051704)	NS	1.7	1.9	2.0	NS	NS	1.8	2.4
Pilus organization (GO:0043711)	2.7	NS	NS	NS	2.8	NS	NS	3.4
Protein secretion (GO:0009306)	NS	NS	NS	NS	NS	NS	NS	2.8
Secretion by cell (GO:0032940)	NS	NS	NS	NS	NS	NS	NS	2.7
Peptide secretion (GO:0002790)	NS	NS	NS	NS	NS	NS	NS	2.8
Transmembrane transport (GO:0055085)	1.4	1.4	1.4	1.4	1.4	1.4	1.4	1.6
Transport (GO:0006810)	1.3	1.3	1.3	1.3	1.3	1.3	1.3	1.4
Establishment of localization (GO:0051234)	1.3	1.3	1.3	1.3	1.3	1.3	1.3	1.4
Localization (GO:0051179)	1.3	1.3	1.3	1.2	1.3	1.2	1.3	1.4

The fold enrichment for genes within each GO term are indicated for each strain and antibiotic treatment compared to the expected values from the reference strain. Only those GO terms that were significantly enriched in at least one strain are included (Bonferroni-correct *P* < 0.05)

*NS* not significantly enriched

### Chlortetracycline induced invasion in two isolates

**Fig. 6:**
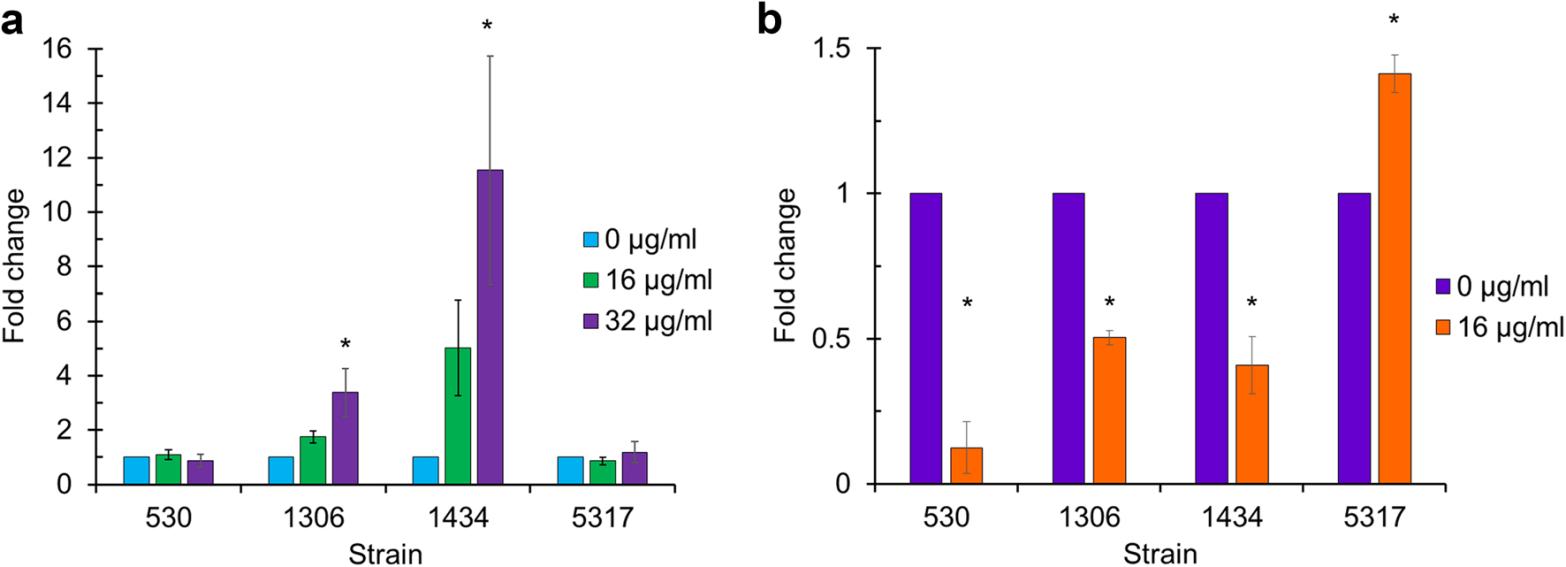
Fold changes in cellular invasion for each MDR *S*. Typhimurium isolate exposed to **a** chlortetracycline at 16 and 32 µg/ml and **b** florfenicol at 16 µg/ml for 30 min. Invasion assays were conducted using HEp-2 cells. Changes in invasion were normalized to the untreated control (0 µg/ml). The asterisk indicates a significantly different invasion rate compared with the control based on pre-normalized data (P < 0.05)

Following exposure to 32 µg/ml of CTC for 30 min, strains 1306 and 1434 both exhibited significantly higher invasion of HEp-2 cells in vitro compared to their respective no-antibiotic controls (Fig. 6a; P < 0.05). However, cellular invasion was not significantly altered in these strains when a CTC concentration of 16 µg/ml was used. The invasion rate of the other two strains, 530 and 5317, was not significantly different at either CTC concentration compared to the no antibiotic control (P > 0.05). Exposure to 16 µg/ml of florfenicol significantly increased invasion of HEp-2 cells for isolate 5317. In contrast, treatment of isolates 530, 1306, and 1434 with florfenicol resulted in a significant decrease in cellular invasion compared to the control in the absence of antibiotic (Fig. 6b; P < 0.05).

The observed changes in SPI-1 gene expression did not necessarily correlate with the invasion phenotype. For example, exposure to florfenicol up-regulated the SPI-1 genes in strains 1434 and 5317, but enhanced invasion was only observed in 5317. In addition, CTC-mediated SPI-1 expression was significantly greater in 1434 and 5317 compared to 530 and 1306, but 1434 and 1306 had induced invasion phenotypes. Furthermore, the innate efflux pumps, such as AcrAB and TolC, have been reported to play a role in *Salmonella* invasion [40], but the expression of the genes encoding these pumps was not consistent with the invasion phenotype of these isolates (Additional file 6: Figure S4).

### Comparison of antibiotic class and invasion following antibiotic exposure

Antibiotic modulation of *Salmonella* invasion is dependent upon the bacterial strain, the antibiotic, and the antibiotic concentration [5, 6]. The invasion phenotype of four MDR *S*. Typhimurium isolates (530, 1306, 1434, and 5317) have been determined following exposure to either tetracycline (1, 4, and 16 µg/ml) [6] or CTC (16 and 32 µg/ml; Fig. 6a). Invasion was only significantly increased for strains 1434, 1306, and 5317 following exposure to tetracycline (16 µg/ml) and CTC (32 µg/ml), CTC (32 µg/ml), and tetracycline (16 µg/ml), respectively. Lower concentrations of tetracycline (1 and 4 µg/ml) or CTC (16 µg/ml) did not significantly increase invasion for any of the MDR *S*. Typhimurium isolates and the higher concentration of tetracycline or CTC only significantly enhanced invasion in certain isolates. The invasion phenotype of MDR *S*. Typhimurium isolates 530, 1306, 1434, and 5317 have also been determined following exposure to either chloramphenicol (16, 32, 64, and 128 µg/ml) [5] or florfenicol (16 µg/ml; Fig. 6b). Invasion was significantly decreased for isolates 1434, 1306, and 530 following exposure to florfenicol (16 µg/ml), florfenicol (16 µg/ml), and chloramphenicol (64 and 128 µg/ml) and florfenicol (16 µg/ml), respectively. Invasion was significantly increased for strains 1434, 1306, and 5317 following exposure to chloramphenicol (32 µg/ml), chloramphenicol (64 µg/ml), and chloramphenicol (64 µg/ml) and florfenicol (16 µg/ml), respectively.

This study further highlights the complexity of *Salmonella* invasion following antibiotic exposure, and the role the underlying genetics of an individual isolate may have on the resulting phenotype. For example, there was a coordinated response for significantly increased gene expression of SPI-1 genes in strain 5317 following CTC exposure (Fig. 3) but invasion was not significantly enhanced (Fig. 6a). Conversely, invasion was significantly enhanced for strain 1306 following CTC exposure but SPI-1 gene expression lacked a robust coordinated response. Exposure to either CTC or florfenicol differentially regulates at least 50% of the genes in MDR *S.* Typhimurium strains that have acquired antimicrobial resistance genes to effectively cope with exposure to these antibiotics. We have evaluated two phenotypic assays (invasion and motility) for *S.* Typhimurium based on coordinated responses in individual regulons (attachment, virulence, and motility). A greater understanding is needed concerning the influence of antibiotic exposure on these individual regulons and the combined contribution of differential gene expression on *Salmonella* physiology. Additional research is also necessary to address survival in the environment and the host for determination of phenotypic advantages and/or disadvantages due to differential gene expression following antibiotic exposure in MDR *S.* Typhimurium.

## Conclusions

The in vitro exposure of four MDR *S*. Typhimurium isolates to either CTC or florfenicol for 30 min resulted in the differential expression of greater than 50% of the genes in the genome. A large number of genes involved in attachment and pathogenicity were enriched following antibiotic exposure. CTC and florfenicol are often administered to livestock for the prevention and treatment of a variety of diseases. Antibiotic treatment of animals that are unknowingly colonized with MDR *S*. Typhimurium may enhance expression of *Salmonella* genes involved in pathogenicity, potentially prolonging host colonization and fecal shedding of this foodborne pathogen. Knowledge of the antibiotics that enhance MDR *Salmonella* virulence mechanisms will provide information to aid in limiting the negative consequences of antibiotic therapy by allowing veterinarians to make informed decisions when determining antibiotic treatment for other bacterial infections.

## Additional files

**Additional file 1: Table S1.** Minimum inhibitory concentrations for each MDR *S*. Typhimurium strain for ampicillin (AMP), chloramphenicol (CPC), chlortetracycline (CTC), florfenicol (FF), streptomycin (STREP), and tetracycline (TET). Values represent µg/ml.

**Additional file 2: Figure S1.** Fold changes for motility-associated genes following exposure to either chlortetracycline at 32 µg/ml or florfenicol at 16 µg/ml for 30 min. Numbers in bold indicate significantly differentially expressed genes (FDR < 0.05). Genes that are up-regulated are colored blue while down-regulated genes are in red; the intensity of the color indicates greater fold change.

**Additional file 3: Figure S2.** Fold changes in A) swarming motility and B) swimming motility for each MDR S. Typhimurium isolate exposed to chlortetracycline at 16 and 32 µg/ml and C) swarming motility and D) swimming motility for florfenicol-treated cells at 16 µg/ml for 30 min. Fold changes in motility were normalized to the untreated control (0 µg/ml). The “*” indicates a significantly different motility compared with the control based on pre-normalized data (P < 0.05). The “#” indicates that no growth was observed in the agar plate.

**Additional file 4: Figure S3.** Fold changes in SOS response genes following exposure to either chlortetracycline at 32 µg/ml or florfenicol at 16 µg/ml for 30 min. Numbers in bold indicate significantly differentially expressed genes (FDR < 0.05). Genes that are up-regulated are colored blue while down-regulated genes are in red; the intensity of the color indicates greater fold change.

**Additional file 5: Table S2.** Gene ontology (GO) terms describing biological processes that were enriched among the significantly differentially underexpressed genes in each *S*. Typhimurium strain and antibiotic treatment. The fold enrichment for genes within each GO term are indicated for each strain and antibiotic treatment compared to the expected values from the reference strain. Only those GO terms that were significantly enriched in at least one strain are included (Bonferroni-correct P-value < 0.05). NS = not significantly enriched.

**Additional file 6: Figure S4.** Fold changes for efflux genes following exposure to either chlortetracycline at 32 µg/ml or florfenicol at 16 µg/ml for 30 min. Numbers in bold indicate significantly differentially expressed genes (FDR < 0.05). Genes that are up-regulated are colored blue while down-regulated genes are in red; the intensity of the color indicates greater fold change.

## Abbreviations

CTC: chlortetracycline
FDR: false discovery rate
GO: gene ontology
MDR: multidrug-resistant
SEM: standard error of the mean
SPI: *Salmonella* pathogenicity island
T3SS: type III secretion system
