# Supplementary material for: Chlortetracycline and florfenicol induce expression of genes associated with pathogenicity in multidrug-resistant Salmonella enterica serovar Typhimurium
Source: Gut Pathog. 2018 Mar 5;10:10. doi: 10.1186/s13099-018-0236-y (PMC5836442; doi:10.1186/s13099-018-0236-y)
Supplement: Supplementary file 1 — Additional file 1: Table S1. Minimum inhibitory concentrations for each MDR S. Typhimurium strain for ampicillin (AMP), chloramphenicol (CPC), chlortetracycline (CTC), florfenicol (FF), streptomycin (STREP), and tetracycline (TET). Values represent µg/ml. [file 13099_2018_236_MOESM1_ESM.docx]

| **Strain** | **Amp** | **CPC** | **CTC** | **FF** | **Strep** | **TET** |
| --- | --- | --- | --- | --- | --- | --- |
| 530 | > 512 | 128 | 128 | 32 | > 512 | 64 |
| 1306 | > 512 | 128 | 256 | 64 | > 512 | 256 |
| 1434 | > 512 | 128 | 256 | 64 | > 512 | 256 |
| 5317 | > 512 | 128 | 256 | 64 | > 512 | 256 |

**Table S1**. Minimum inhibitory concentrations for each *S*. Typhimurium strain for ampicillin (AMP), chloramphenicol (CPC), chlortetracycline (CTC), florfenicol (FF), streptomycin (STREP), and tetracycline (TET). Values represent µg/ml.
