# Supplementary figures and images for: Chlortetracycline and florfenicol induce expression of genes associated with pathogenicity in multidrug-resistant Salmonella enterica serovar Typhimurium
Source: Gut Pathog. 2018 Mar 5;10:10. doi: 10.1186/s13099-018-0236-y (PMC5836442; doi:10.1186/s13099-018-0236-y)

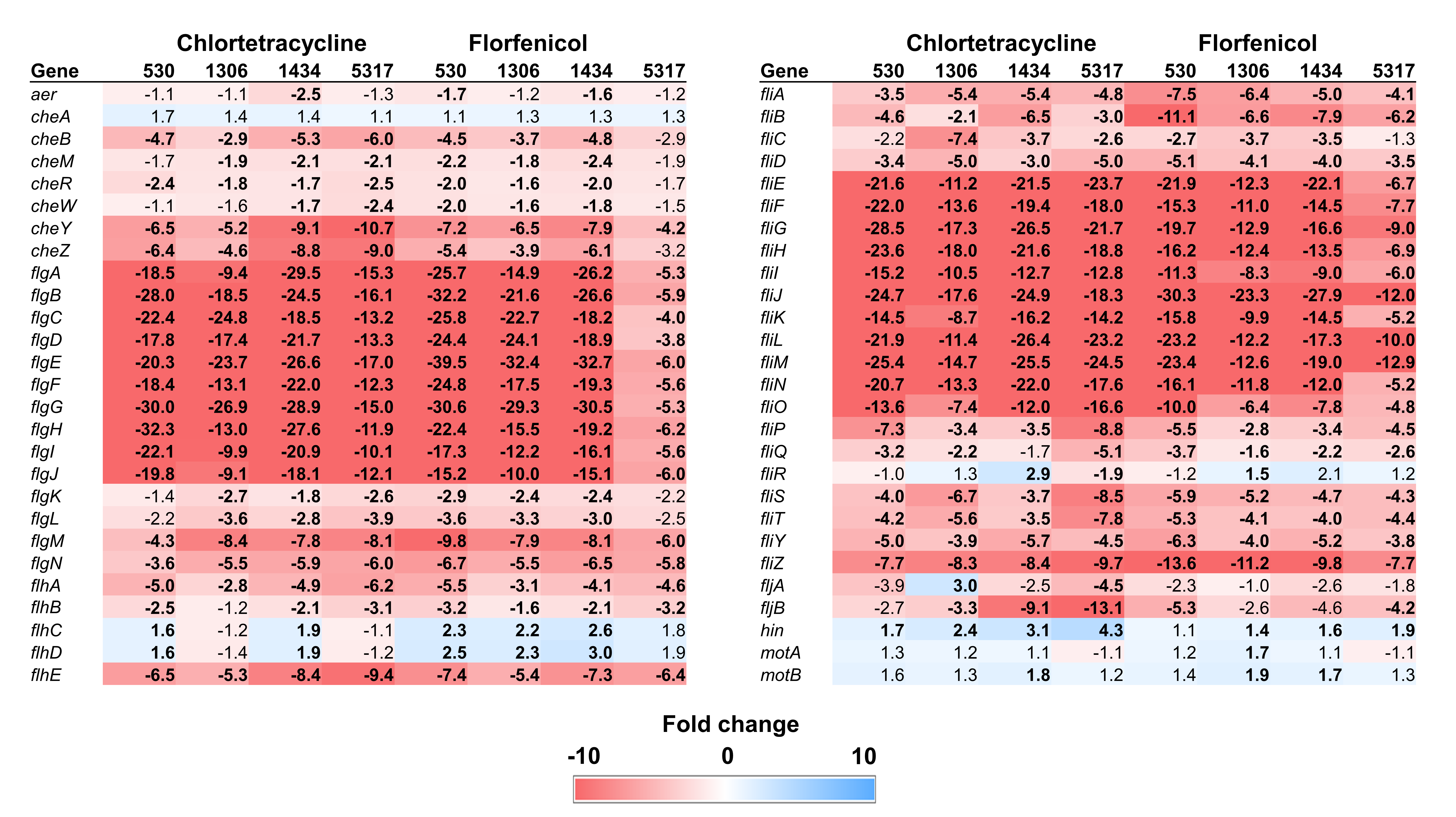

Supplement: Supplementary file 2 — Additional file 2: Figure S1. Fold changes for motility-associated genes following exposure to either chlortetracycline at 32 µg/ml or florfenicol at 16 µg/ml for 30 min. Numbers in bold indicate significantly differentially expressed genes (FDR < 0.05). Genes that are up-regulated are colored blue while down-regulated genes are in red; the intensity of the color indicates greater fold change. [file 13099_2018_236_MOESM2_ESM.tif]

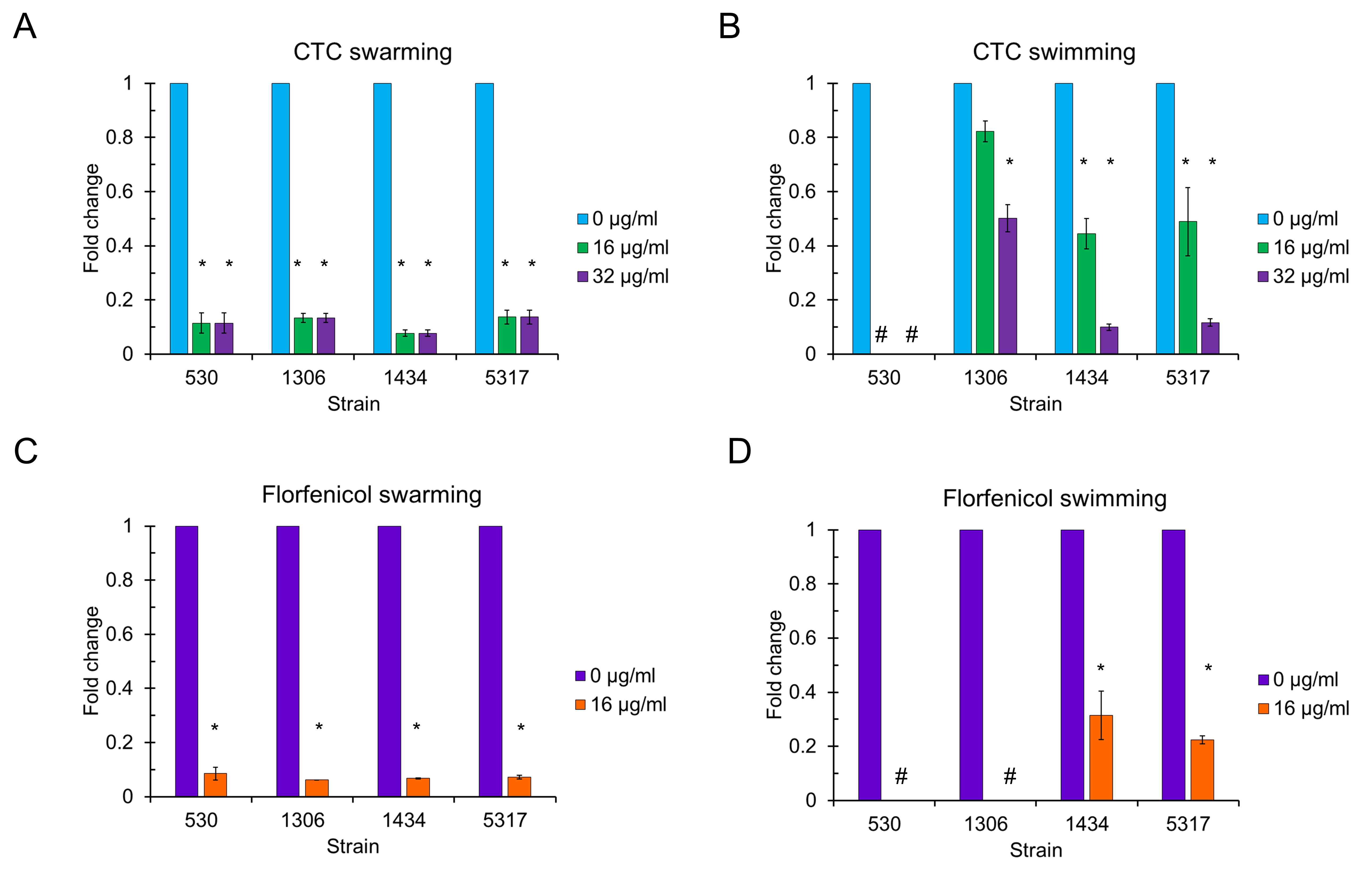

Supplement: Supplementary file 3 — Additional file 3: Figure S2. Fold changes in A) swarming motility and B) swimming motility for each MDR S. Typhimurium isolate exposed to chlortetracycline at 16 and 32 µg/ml and C) swarming motility and D) swimming motility for florfenicol-treated cells at 16 µg/ml for 30 min. Fold changes in motility were normalized to the untreated control (0 µg/ml). The “*” indicates a significantly different motility compared with the control based on pre-normalized data (P < 0.05). The “#” indicates that no growth was observed in the agar plate. [file 13099_2018_236_MOESM3_ESM.tif]

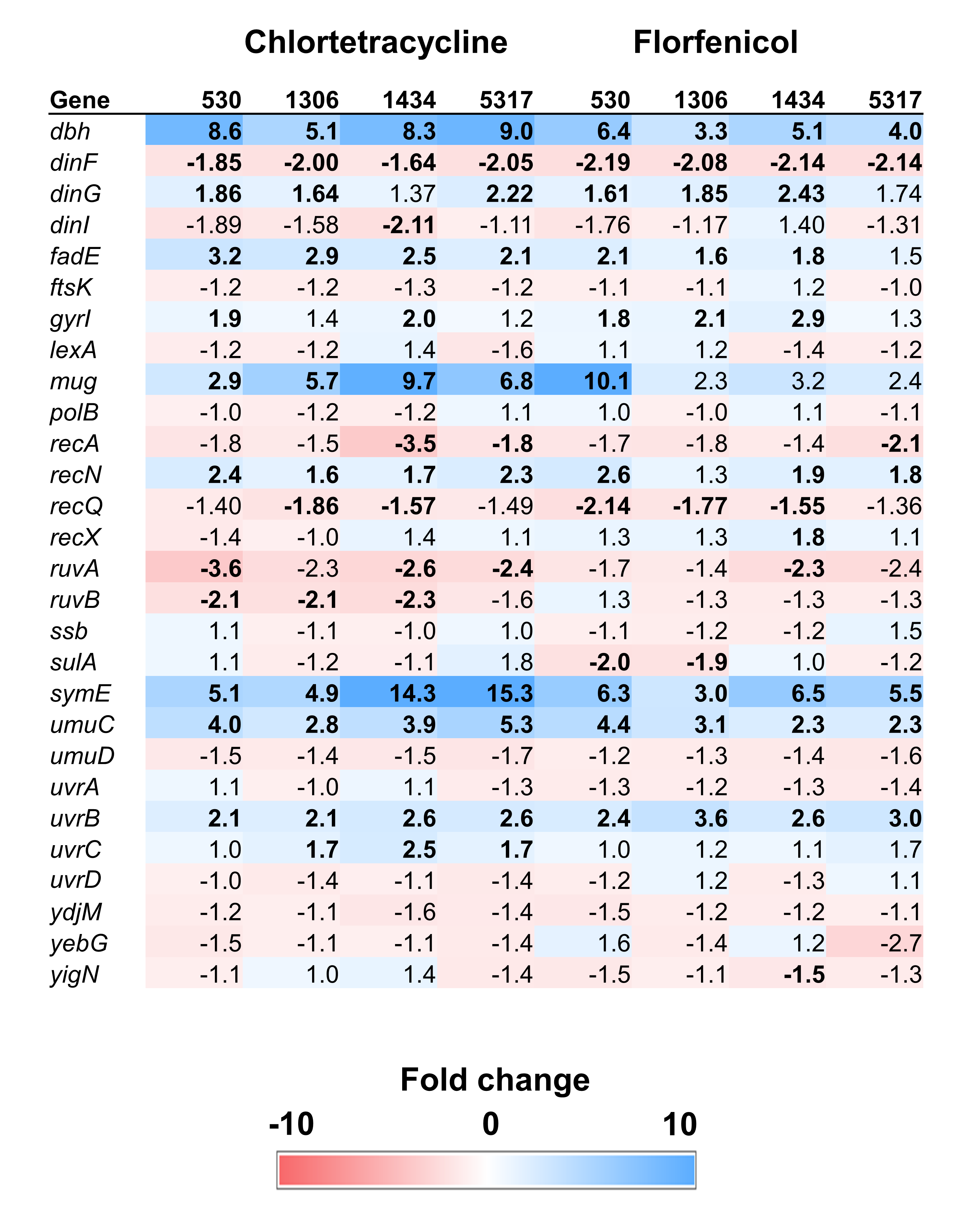

Supplement: Supplementary file 4 — Additional file 4: Figure S3. Fold changes in SOS response genes following exposure to either chlortetracycline at 32 µg/ml or florfenicol at 16 µg/ml for 30 min. Numbers in bold indicate significantly differentially expressed genes (FDR < 0.05). Genes that are up-regulated are colored blue while down-regulated genes are in red; the intensity of the color indicates greater fold change. [file 13099_2018_236_MOESM4_ESM.tif]

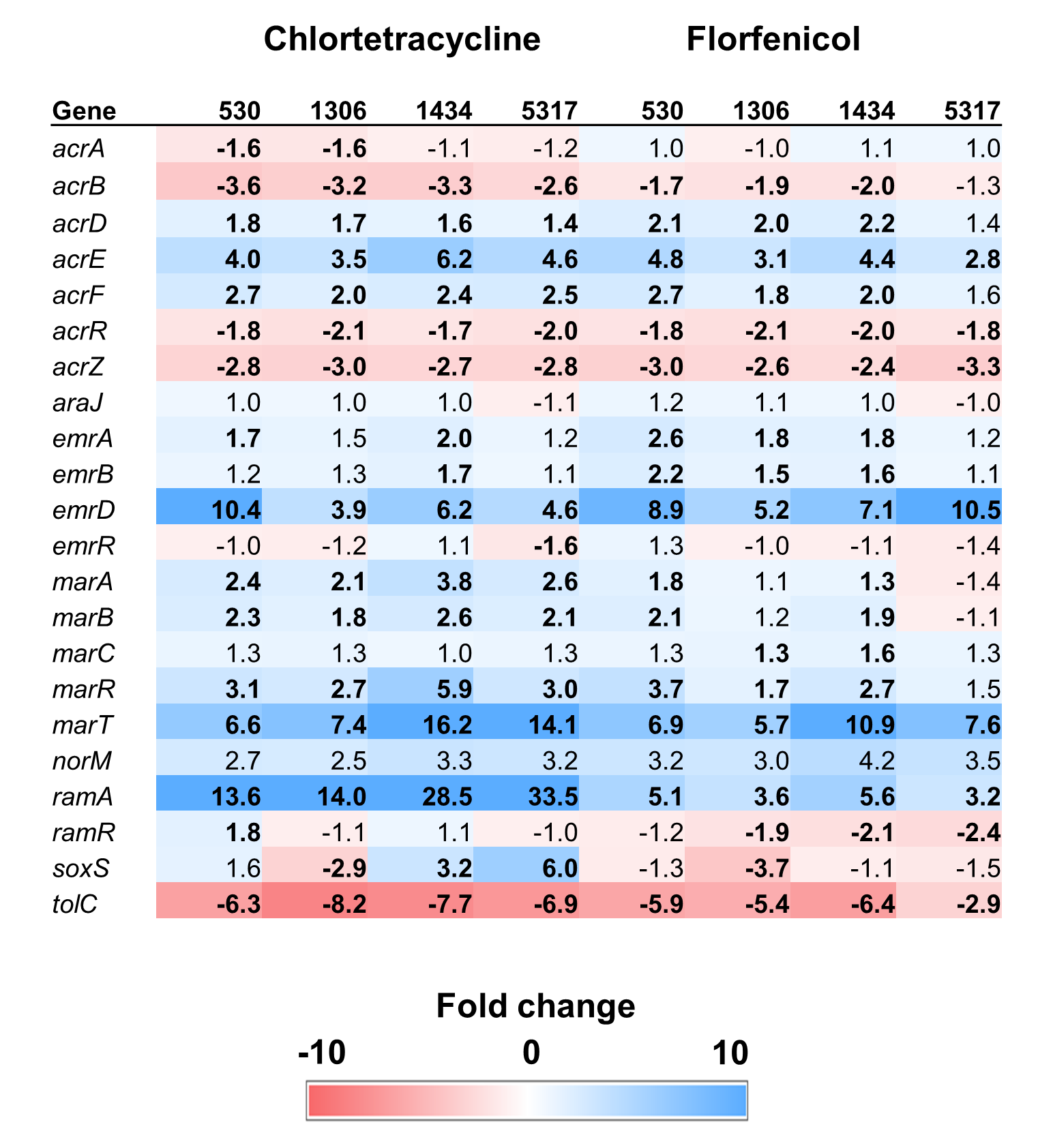

Supplement: Supplementary file 6 — Additional file 6: Figure S4. Fold changes for efflux genes following exposure to either chlortetracycline at 32 µg/ml or florfenicol at 16 µg/ml for 30 min. Numbers in bold indicate significantly differentially expressed genes (FDR < 0.05). Genes that are up-regulated are colored blue while down-regulated genes are in red; the intensity of the color indicates greater fold change. [file 13099_2018_236_MOESM6_ESM.tif]
