# Supplementary material for: Chlortetracycline and florfenicol induce expression of genes associated with pathogenicity in multidrug-resistant Salmonella enterica serovar Typhimurium
Source: Gut Pathog. 2018 Mar 5;10:10. doi: 10.1186/s13099-018-0236-y (PMC5836442; doi:10.1186/s13099-018-0236-y)
Supplement: Supplementary file 5 — Additional file 5: Table S2. Gene ontology (GO) terms describing biological processes that were enriched among the significantly differentially underexpressed genes in each S. Typhimurium strain and antibiotic treatment. The fold enrichment for genes within each GO term are indicated for each strain and antibiotic treatment compared to the expected values from the reference strain. Only those GO terms that were significantly enriched in at least one strain are included (Bonferroni-correct P-value < 0.05). NS = not significantly enriched. [file 13099_2018_236_MOESM5_ESM.docx]

**Table S2**. Gene ontology (GO) terms describing biological processes that were enriched among the differentially underexpressed genes in each *S*. Typhimurium strain and antibiotic treatment. The fold enrichment for genes within each GO term are indicated for each strain and antibiotic treatment compared to the expected values from the reference strain. Only those GO terms with that were significantly enriched in at least one strain are included (Bonferroni-correct P-value < 0.05). NS = not significantly enriched.

|  | **Chlortetracycline** | | | | **Florfenicol** | | | |
| --- | --- | --- | --- | --- | --- | --- | --- | --- |
| **Gene ontology term** | **530** | **1306** | **1434** | **5317** | **530** | **1306** | **1434** | **5317** |
| Bacterial-type flagellum organization (GO:0044781) | NS | NS | NS | NS | NS | NS | NS | 4.0 |
| Bacterial-type flagellum-dependent cell motility (GO:0071973) | NS | NS | NS | NS | NS | NS | NS | 3.8 |
| Archaeal or bacterial-type flagellum-dependent cell motility (GO:0097588) | NS | 2.5 | 2.5 | NS | 2.5 | NS | NS | 3.6 |
| Cilium or flagellum-dependent cell motility (GO:0001539) | NS | 2.5 | 2.5 | NS | 2.5 | NS | NS | 3.6 |
| Cell motility (GO:0048870) | NS | 2.5 | 2.5 | NS | 2.5 | NS | NS | 3.6 |
| Movement of cell or subcellular component (GO:0006928) | NS | 2.5 | 2.5 | NS | 2.5 | NS | NS | 3.6 |
| Single-organism cellular process (GO:0044763) | NS | 1.4 | 1.5 | NS | 1.4 | NS | NS | NS |
| Single-organism process (GO:0044699) | NS | 1.3 | 1.4 | NS | 1.3 | NS | NS | NS |
| Localization of cell (GO:0051674) | NS | 2.5 | 2.5 | NS | 2.5 | NS | NS | 3.6 |
| ATP metabolic process (GO:0046034) | 2.4 | NS | 2.4 | NS | NS | NS | NS | 3.2 |
| Purine ribonucleoside triphosphate metabolic process (GO:0009205) | 2.4 | NS | 2.4 | NS | 2.3 | NS | NS | 3.0 |
| Ribonucleoside triphosphate metabolic process (GO:0009199) | NS | NS | NS | NS | NS | NS | NS | 2.7 |
| Nucleoside triphosphate metabolic process (GO:0009141) | 2.1 | NS | NS | NS | NS | NS | NS | NS |
| Nucleoside phosphate metabolic process (GO:0006753) | 1.7 | 1.7 | NS | 1.8 | NS | 1.6 | 1.7 | 2.0 |
| Organophosphate metabolic process (GO:0019637) | 1.6 | 1.7 | 1.5 | 1.6 | 1.5 | 1.5 | 1.5 | 1.7 |
| Phosphorus metabolic process (GO:0006793) | 1.4 | 1.4 | 1.4 | 1.4 | 1.3 | 1.3 | 1.4 | NS |
| Cellular metabolic process (GO:0044237) | 1.3 | 1.3 | 1.3 | 1.2 | 1.2 | 1.2 | 1.2 | 1.2 |
| Cellular process (GO:0009987) | 1.3 | 1.3 | 1.2 | 1.2 | 1.2 | 1.2 | 1.2 | 1.2 |
| Metabolic process (GO:0008152) | 1.3 | 1.3 | 1.3 | 1.3 | 1.2 | 1.2 | 1.3 | 1.2 |
| Organic substance metabolic process (GO:0071704) | 1.3 | 1.3 | 1.3 | 1.3 | 1.2 | 1.2 | 1.2 | 1.2 |
| Phosphate-containing compound metabolic process (GO:0006796) | 1.4 | 1.4 | 1.4 | 1.4 | 1.3 | 1.4 | 1.4 | 1.5 |
| Nucleobase-containing small molecule metabolic process (GO:0055086) | 1.6 | 1.6 | NS | 1.6 | NS | 1.6 | 1.6 | NS |
| Organic cyclic compound metabolic process (GO:1901360) | 1.2 | 1.3 | NS | 1.2 | NS | NS | 1.2 | NS |
| Nitrogen compound metabolic process (GO:0006807) | 1.2 | 1.2 | 1.2 | 1.2 | NS | 1.2 | 1.2 | 1.2 |
| Heterocycle metabolic process (GO:0046483) | 1.2 | 1.3 | NS | 1.3 | NS | 1.2 | 1.2 | NS |
| Cellular aromatic compound metabolic process (GO:0006725) | 1.2 | 1.2 | NS | 1.2 | NS | NS | NS | NS |
| Primary metabolic process (GO:0044238) | 1.3 | 1.3 | 1.2 | 1.2 | 1.2 | 1.2 | 1.2 | 1.2 |
| Single-organism cellular process (GO:0044763) | 1.5 | 1.4 | NS | NS | NS | 1.2 | NS | NS |
| Single-organism process (GO:0044699) | 1.4 | 1.3 | NS | NS | NS | NS | NS | NS |
| Small molecule metabolic process (GO:0044281) | 1.6 | 1.5 | 1.6 | 1.5 | 1.4 | 1.4 | 1.5 | 1.3 |
| Single-organism metabolic process (GO:0044710) | 1.5 | 1.4 | 1.5 | NS | NS | NS | NS | 1.4 |
| Purine nucleoside triphosphate metabolic process (GO:0009144) | 2.3 | NS | NS | NS | NS | NS | NS | 2.9 |
| Purine ribonucleoside monophosphate metabolic process (GO:0009167) | NS | NS | NS | NS | NS | NS | NS | 2.6 |
| Purine nucleoside monophosphate metabolic process (GO:0009126) | NS | NS | NS | NS | NS | NS | NS | 2.6 |
| Purine ribonucleotide metabolic process (GO:0009150) | 2.1 | 1.9 | 2.3 | 1.9 | NS | NS | 1.9 | 2.3 |
| Purine nucleotide metabolic process (GO:0006163) | 1.9 | NS | NS | NS | NS | NS | 1.8 | NS |
| Purine-containing compound metabolic process (GO:0072521) | 1.9 | 1.9 | NS | 1.8 | NS | NS | 1.8 | NS |
| Organonitrogen compound metabolic process (GO:1901564) | 1.4 | 1.3 | 1.3 | 1.3 | 1.2 | 1.3 | 1.3 | NS |
| Nucleotide metabolic process (GO:0009117) | 1.7 | 1.6 | NS | 1.7 | NS | 1.6 | 1.7 | 2.0 |
| Ribose phosphate metabolic process (GO:0019693) | 1.9 | NS | NS | 1.8 | NS | NS | 1.8 | 2.1 |
| Carbohydrate derivative metabolic process (GO:1901135) | 1.6 | 1.6 | 1.5 | 1.6 | 1.4 | 1.5 | 1.5 | 1.6 |
| Nicotinamide nucleotide metabolic process (GO:0046496) | NS | NS | NS | NS | NS | NS | NS | 3.1 |
| Pyridine nucleotide metabolic process (GO:0019362) | NS | NS | NS | NS | NS | NS | NS | 3.1 |
| Pyridine-containing compound metabolic process (GO:0072524) | NS | NS | NS | NS | NS | NS | NS | 3.0 |
| Tetrapyrrole biosynthetic process (GO:0033014) | 2.4 | 2.2 | 2.3 | 2.3 | NS | 2.2 | NS | NS |
| Cellular biosynthetic process (GO:0044249) | 1.3 | 1.3 | 1.2 | 1.3 | 1.2 | 1.2 | 1.3 | 1.3 |
| Biosynthetic process (GO:0009058) | 1.3 | 1.3 | 1.2 | 1.3 | 1.2 | 1.2 | 1.3 | 1.3 |
| Organic substance biosynthetic process (GO:1901576) | 1.3 | 1.3 | 1.2 | 1.3 | 1.2 | 1.2 | 1.3 | 1.3 |
| Tetrapyrrole metabolic process (GO:0033013) | 2.4 | 2.2 | 2.3 | 2.3 | NS | 2.2 | 2.1 | NS |
| Cellular respiration (GO:0045333) | 2.1 | 2.0 | 2.2 | NS | NS | NS | NS | NS |
| Energy derivation by oxidation of organic compounds (GO:0015980) | 2.2 | 2.1 | 2.3 | NS | NS | 1.9 | 1.9 | NS |
| Generation of precursor metabolites and energy (GO:0006091) | 2.2 | 2.1 | 2.2 | 1.8 | 1.7 | 1.9 | 1.9 | 2.2 |
| Oxidation-reduction process (GO:0055114) | 1.5 | 1.4 | 1.6 | 1.4 | 1.4 | 1.4 | 1.5 | 1.5 |
| Coenzyme metabolic process (GO:0006732) | 1.7 | 1.7 | 1.7 | 1.7 | NS | NS | NS | NS |
| Cofactor metabolic process (GO:0051186) | 1.6 | 1.6 | 1.6 | 1.6 | NS | NS | NS | NS |
| Cellular polysaccharide metabolic process (GO:004426) | 1.7 | 1.8 | NS | 1.7 | 1.6 | 1.7 | NS | NS |
| Polysaccharide metabolic process (GO:0005976) | NS | 1.7 | NS | NS | NS | NS | NS | NS |
| Carbohydrate metabolic process (GO:0005975) | 1.5 | 1.5 | 1.6 | 1.5 | 1.6 | 1.5 | 1.5 | 1.6 |
| Cellular carbohydrate metabolic process (GO:0044262) | NS | 1.6 | 1.6 | NS | 1.6 | 1.6 | 1.6 | NS |
| Cellular catabolic process (GO:0044248) | NS | NS | 1.7 | NS | 1.6 | 1.6 | 1.5 | NS |
| Polysaccharide biosynthetic process (GO:0000271) | NS | 1.8 | NS | NS | NS | NS | NS | 2.1 |
| Carbohydrate biosynthetic process (GO:0016051) | NS | 1.6 | NS | NS | NS | NS | NS | 1.9 |
| Single-organism biosynthetic process (GO:0044711) | NS | 1.5 | 1.5 | NS | NS | NS | NS | NS |
| Cellular carbohydrate biosynthetic process (GO:0034637) | NS | 1.7 | NS | NS | 1.7 | NS | NS | 2.1 |
| alpha-Amino acid metabolic process (GO:1901605) | 1.6 | NS | 1.6 | NS | NS | NS | NS | NS |
| Cellular amino acid metabolic process (GO:0006520) | NS | 1.5 | NS | 1.4 | 1.5 | NS | 1.5 | NS |
| Carboxylic acid metabolic process (GO:0019752) | NS | 1.5 | NS | NS | NS | 1.5 | 1.5 | NS |
| Oxoacid metabolic process (GO:0043436) | NS | 1.5 | NS | NS | NS | NS | 1.5 | 1.4 |
| Organic acid metabolic process (GO:0006082) | 1.6 | 1.5 | 1.4 | 1.5 | 1.4 | 1.4 | 1.5 | 1.5 |
| Cellular polysaccharide biosynthetic process (GO:0033692) | NS | 1.8 | NS | NS | NS | NS | NS | 2.1 |
| Response to chemical (GO:0042221) | NS | NS | NS | NS | NS | NS | NS | 2.0 |
| Macromolecule metabolic process (GO:0043170) | NS | 1.2 | NS | NS | NS | NS | NS | NS |
| Heterocycle biosynthetic process (GO:0018130) | NS | 1.3 | NS | 1.3 | 1.3 | 1.3 | 1.3 | NS |
| Monocarboxylic acid metabolic process (GO:0032787) | NS | NS | 1.7 | NS | NS | NS | 1.7 | NS |
| Organic cyclic compound biosynthetic process (GO:1901362) | NS | 1.3 | NS | 1.3 | 1.3 | 1.3 | 1.3 | NS |
| Organic substance catabolic process (GO:1901575) | NS | NS | 1.6 | 1.5 | 1.4 | NS | 1.4 | NS |
| Oxidation-reduction process (GO:0055114) | NS | 1.4 | 1.6 | 1.4 | 1.4 | 1.4 | NS | NS |
| Single-organism carbohydrate metabolic process (GO:0044723) | 1.5 | 1.5 | 1.6 | NS | 1.5 | 1.5 | 1.6 | NS |
| Single-organism catabolic process (GO:0044712) | 1.5 | NS | 1.8 | NS | NS | NS | NS | NS |
| Small molecule biosynthetic process (GO:0044283) | 1.5 | 1.4 | 1.5 | NS | NS | NS | NS | NS |
| Carbohydrate derivative biosynthetic process (GO:1901137) | 1.5 | 1.5 | NS | 1.6 | NS | NS | 1.5 | NS |
| Nucleoside monophosphate metabolic process (GO:0009123) | NS | NS | NS | 1.9 | NS | NS | 1.8 | NS |
| Ribonucleotide metabolic process (GO:0009259) | NS | NS | NS | 1.8 | NS | NS | 1.8 | NS |
| Cellular polysaccharide metabolic process (GO:0044264) | 1.7 | 1.8 | NS | 1.7 | NS | 1.7 | 1.7 | 2.0 |
| Response to stimulus (GO:0050896) | NS | NS | NS | NS | NS | NS | NS | 1.5 |
| Catabolic process (GO:0009056) | NS | NS | 1.6 | 1.4 | 1.4 | NS | 1.4 | NS |
| Organonitrogen compound biosynthetic process (GO:1901566) | NS | NS | NS | 1.3 | NS | NS | 1.3 | NS |
| Aromatic compound biosynthetic process (GO:0019438) | NS | 1.3 | NS | NS | NS | 1.3 | 1.3 | NS |
| Biological_process (GO:0008150) | 1.1 | 1.1 | 1.1 | 1.1 | 1.1 | 1.1 | 1.1 | 1.1 |
